# Benznidazole and amiodarone combined treatment attenuates cytoskeletal damage in *Trypanosoma cruzi*-infected cardiac cells

**DOI:** 10.3389/fcimb.2022.975931

**Published:** 2022-08-25

**Authors:** Juliana Magalhães Chaves Barbosa, Yasmin Pedra-Rezende, Luíza Dantas Pereira, Tatiana Galvão de Melo, Helene Santos Barbosa, Joseli Lannes-Vieira, Solange Lisboa de Castro, Anissa Daliry, Kelly Salomão

**Affiliations:** ^1^ Laboratório de Biologia Celular, Instituto Oswaldo Cruz, Rio de Janeiro, Brazil; ^2^ Laboratório de Ultraestrutura Celular, Instituto Oswaldo Cruz, Rio de Janeiro, Brazil; ^3^ Laboratóriode de Biologia Estrutural, Instituto Oswaldo Cruz, Rio de Janeiro, Brazil; ^4^ Laboratório de Biologia das Interações, Instituto Oswaldo Cruz, Rio de Janeiro, Brazil; ^5^ Laboratório de Investigação Cardiovascular, Instituto Oswaldo Cruz, Rio de Janeiro, Brazil

**Keywords:** *Trypanosoma cruzi*, Chagas disease, amiodarone, benznidazole, combined treatment

## Abstract

Chagas disease (CD), a neglected tropical disease caused by the protozoan parasite *Trypanosoma cruzi*, is an important public health problem mainly in Latin America, leading to approximately 12,000 annual deaths. Current etiological treatment for CD is limited to two nitro compounds, benznidazole (Bz) and nifurtimox (Nif), both presenting relevant limitations. Different approaches have been employed to establish more effective and safer schemes to treat *T. cruzi* infection, mostly based on drug repurposing and combination therapies. Amiodarone (AMD), an antiarrhythmic medicament of choice for patients with the chronic cardiac form of CD, is also recognized as a trypanocidal agent. Therefore, our aim is to investigate the combined treatment Bz + AMD on trypomastigote viability, control of *T. cruzi* intracellular form proliferation, and recovery of the infection-induced cytoskeleton alterations in cardiac cells. The combination of Bz + AMD did not improve the direct trypanocidal effect of AMD on the infective blood trypomastigote and replicative intracellular forms of the parasite. Otherwise, the treatment of *T. cruzi*-infected cardiac cells with Bz plus AMD attenuated the infection-triggered cytoskeleton damage of host cells and the cytotoxic effects of AMD. Thus, the combined treatment Bz + AMD may favor parasite control and hamper tissue damage.

## Introduction

Chagas disease (CD) is caused by the protozoan *Trypanosoma cruzi*, an important public health problem endemic of Latin America, that affects approximately 6-8 million of people worldwide, causing nearly 12,000 annual deaths (PAHO, 2018; Lidani et al., 2019). In recent decades, due to increasing global migration, CD patients have lived in nonendemic places, such as North America, Europe, Australia and Japan (Pérez-Molina and Molina, 2018). The transmission of this protozoan is primarily through contact with feces of triatomine insects after biting. Furthermore, transmission can also occur *via* non-vectorial route, by ingestion of contaminated food, congenitally and through blood transfusion or organ transplantation (Antunes et al., 2019).

The natural clinical course of CD comprises two phases: acute and chronic (Chatelain, 2015). The acute phase of DC, occurs within the first weeks after parasite infection, is characterized by high parasitemia and is frequently asymptomatic. Nonetheless, this phase may present mild flu-like nonspecific symptoms or signs of portal of entry as: chagoma (cutaneous lesion), Romaña sign (palpebral oedema) (Bern, 2011; Pérez-Molina and Molina, 2018). About 2-3 months after infection, untreated patients progress from an acute to a chronic phase, characterized by immune-mediated parasite control, leaving approximately 60-70% of the cases in the indeterminate stage, while the other 30-40%, over the decades (10-30 years), develop clinical signs characteristic of a cardiac and/or digestive pathology (Rassi et al., 2010; Echeverria and Morillo, 2019).

Current etiological treatment for CD is limited to two nitro compounds, benznidazole (Bz) and nifurtimox (Nif), and both present relevant limitations including the occurrence of resistant strains, the lack of efficacy in the later chronic phase, with low cure rates (8 - 30%) and side effects as: weight loss, nausea, headache and allergic dermatitis (Bern, 2011; Ribeiro et al., 2020). Different therapeutic approaches are being used to identify more effective and safer treatment schemes, mostly using drug repurposing and combination strategies (Miranda and Sayé, 2019). Drug combination allows the reduction of doses, costs and time of treatment. In addition, this strategy may overcome the natural or acquired resistance of parasites because the use of molecules with different mechanisms of action could aim more than one target simultaneously (Mazzeti et al., 2021). Drug repurposing is particularly relevant for neglected diseases because this approach reduces the time and budget in the drug discovery process (Ashburn and Thor, 2004).

Amiodarone (AMD) is the most widely used drug for CD patients with cardiac arrhythmia (Dias et al., 2016; Brasil. Ministério da Saúde. Comissão Nacional de Incorporação de Tecnologias no SUS, 2018). In addition, AMD repurposing has been proposed because the trypanocidal activity of this drug has already been established in both *in vitro* and *in vivo* studies, reducing the parasitemia peak in experimentally infected mice (Benaim et al., 2006; Benaim and PanizMondolfi, 2012). The *in vitro* cardioprotective effect of AMD was also described using *T. cruzi*-infected cardiomyocytes, which had recovered spontaneous contractility and the expression of actin filaments and connexin-43 (Adesse et al., 2011).

Recently, our group reported, in a well-established murine model of acute CD, that the combination Bz + AMD was more effective in reducing the peak parasitemia than each drug separated. Additionally, such combination led to the improvement of atrial function, reduction of interleukin-6 and restoration of gap junction integrity in cardiac tissue (Barbosa et al., 2022). Previously, the BENEFIT study showed that Bz treatment did not prevent the progression of chronic chagasic cardiomyopathy (CCC), but patients treated concomitantly with Bz and AMD presented a reduction in both hospitalization incidence and risk of death due to cardiovascular complications (Bz and AMD *versus* placebo, *p*-value: 0.008) (Morillo et al., 2015; Rassi et al., 2017).

Based on the above-mentioned evidence, we hypothesized that the improvement in prognosis observed in an experimental acute model of *T. cruzi* infection and patients with CCC could be related to an augmentation of the trypanocidal activity and to the heart cell cytoskeleton architecture recovery exerted by the combination Bz + AMD. To study these possible effects, we established primary cultures of cardiac cells and *in vitro* infection protocols, allowing analyses of the combined treatment against the infective forms of *T. cruzi* using a fixed-ratio method and host cell morphological analysis by electron microscopy and immunofluorescence.

## Materials and methods

### Compounds

Stock solutions of 100 mM Bz (*N*-benzyl-2-nitro-1*H*-imidazole-1-acetamide; Sigma Aldrich™, St Louis, USA) and AMD (2-butyl-3-benzofuranyl-4-[2-(diethylamino) ethoxy]-3,5-diiodophenyl ketone hydrochloride; Sigma™) were prepared in dimethyl sulfoxide (DMSO; Sigma™), and aliquots were stored at -20°C. The final concentration of the solvent in the assay never exceeded 0.6%, which does not exert any toxicity (Araujo-Lima et al., 2018).

### Parasite

Bloodstream trypomastigotes (BT) of the Y strain of *T. cruzi* (DTU II) were obtained by cardiac puncture of infected Swiss Webster mice at the parasitemia peak by differential centrifugation (500 × g for 30 min at 4°C). The parasites were resuspended in RPMI-1640 medium (Life Technologies™, São Paulo, Brazil) supplemented with 10% mycoplasma-free and inactivated fetal bovine serum (FBS; Cultilab™, São Paulo, Brazil), 1 mM L-glutamine (Sigma™) and 1% penicillin/streptomycin solution (Life Technologies™). The Y strain was previously classified as a partially resistant to treatment with Bz, exhibit high virulence and may produce cardiac disease and mega syndromes (Filardi and Brener, 1987; Martinez et al., 2020).

### Activity against bloodstream forms of *T. cruzi*

For the monotreatment assays, BT (5×10^6^ cells/mL) were incubated at 37°C in a 5% CO_2_ atmosphere in the absence or presence of Bz or AMD at serial concentrations up to 80 µM. After incubation for 24 h, cell counts were performed in a Neubauer chamber by light microscopy (Zeiss™, Oberkochen, Germany), and the activity of the compounds was expressed as the IC_50_/24 h, corresponding to the concentration that led to 50% lysis of the parasites. The combined treatment with Bz and AMD was analyzed using a fixed-ratio method described by Fivelman et al. (2004). The IC_50_ values of Bz and AMD in single treatment were used to establish the top concentrations, ensuring that IC_50_ fell near the midpoint of a six-point twofold dilution series using fixed-ratio solutions: 5:0, 4:1, 3:2, 2:3 and 1:4 proportions. The nature of the interaction was measured based on the fractional inhibitory concentrations (FICs) and on the sum of FICs (ΣFICs) of each compound. The FIC of AMD was calculated as follows: IC_50_ of AMD in combination/IC_50_ of AMD in monotreatment. The same equation was applied to Bz. The ΣFICs = FIC(AMD) + FIC(Bz). An overall ΣFICs was determined and used to classify the nature of each interaction, with ΣFICs ≤ 0.5 = synergism, 0.5 < ΣFICs ≤ 4.0 = additive (no interaction) and ΣFICs > 4.0 = antagonism. Isobolograms were built by plotting the FIC of AMD against the FIC of Bz (Odds, 2003; Simões-Silva et al., 2016).

### Activity against intracellular forms of *T. cruzi* and trypomastigotes release

**Figure 1:**
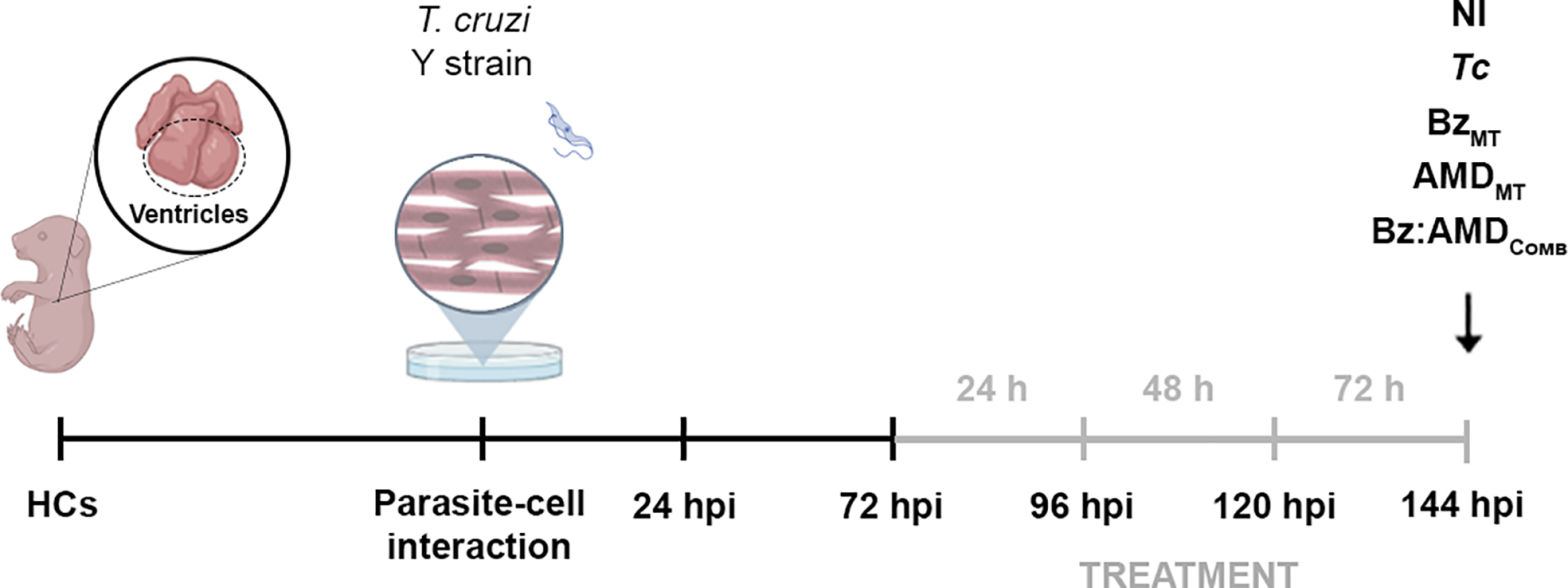
Experimental design of the treatment with Bz, AMD or their combination of *T. cruzi*-infected mouse embryo heart cells (HCs). Cell cultures were exposed to BT of *T. cruzi* for 24 h and then washed to remove noninternalized parasites. Treatment started at 72 hpi and was performed for 24, 48 and 72 h (gray line). Treatment for 72 h was defined for the combined therapy assays. At this time (144 hpi), HCs *T. cruzi*-infected (*Tc*) or non-infected (NI) were treated with the compounds at concentrations corresponding to the IC_50_/72 h in monotreatment (Bz_MT_ and AMD_MT_) and in combination at a ratio of 1:4 (Bz:AMD_Comb_). The illustration is created with *Biorender.com*.

Evaluation of the activity of AMD and Bz against intracellular forms was performed using primary cultures of 18-day-old mouse embryo heart cells (HCs) (Meirelles et al., 1986). The HCs were obtained, as reported by Freitas et al. (2020), cultivated in Dulbecco’s modified Eagle medium (DMEM; Life Technologies™) containing 10% fetal bovine serum (FBS; Cultilab™), 2.5 mM CaCl_2_ (Sigma™), 1 mM L-glutamine (Sigma™), 2% chicken embryo extract and 1% penicillin/streptomycin solution (Life Technologies™), plated in 24-well plates at a density of 1.5x10^5^ cells/well in glass coverslips coated with 0.01% gelatin (Sigma™) and maintained at 37°C in a 5% CO_2_ atmosphere. HCs were infected with BT (MOI [multiplicity of infection]: 10:1, parasites/host cells) in a final volume of 500 μL supplemented DMEM-FBS. After 24 h, the cultures were washed with phosphate buffer, 1X (PBS; Sigma™) to remove nonadherent parasites and maintained in supplemented DMEM-FBS for 72 h postinfection (hpi) before starting the treatment with the drugs. For the monotreatment assays (groups Bz_MT_ and AMD_MT_), infected HCs were incubated for 24, 48 and 72 h at 37°C in a 5% CO_2_ atmosphere in the absence or presence of the compounds in serially diluted nontoxic concentrations (up to 20 µM). The culture medium with or without the drugs was replaced daily, maintaining a total volume of 1 mL in each well. After 24, 48 and 72 h of treatment, the cultures were rinsed with saline, fixed and stained with Diff-Quick Staining (Laborclin™, Paraná, Brazil). The percentage of infection was quantified by randomly counting at least 200 cells per coverslip and examined by light microscopy. In addition, supernatants were collected, and released parasites were counted daily in a Neubauer chamber. The result was expressed by the infection index (II), which corresponds to the multiplication of the percentage of infection by the number of parasites/infected cells (Freitas et al., 2020). The IC_50_ values were calculated for the different days of treatment, corresponding to the concentration that led to 50% inhibition of this parameter (IC_50_ II). The combined treatment of Bz and AMD (Bz:AMD_Comb_) was analyzed after 72 h of treatment using a fixed-ratio method, as mentioned above (Fivelman et al., 2004) (**Figure 1**).

### Mammal cytotoxicity evaluation

Non-infected HCs were incubated at 37°C for 72 h with increasing concentrations of Bz and AMD (2.5 to 20 mM; 1:2 serial dilutions) in monotreatment and combination. After treatment, PrestoBlue™ (Invitrogen™, Life Technologies, USA) was added at a ratio of 1:10, the microplates were incubated for 2 h, and the fluorescence was measured at 560 and 590 nm, as recommended by the manufacturer, using a Spectra Max™ M3 spectrofluorometer (Molecular Devices™, Sunnyvale, EUA). The results were expressed as a percentage of viability using the untreated cells as a reference.

### Immunofluorescence

*T. cruzi*-infected HCs were treated for 72 h with the compounds at the concentrations corresponding to the IC_50_/72 h in single treatment or in combination. Cells were fixed for 20 min at 4°C with 4% paraformaldehyde (Sigma™) in PBS. Actin filaments (F-actin) were visualized with AlexaFluor 488-labeled phalloidin™ (Thermo Fisher Scientific™, Waltham, USA), and DNA was detected with 4’,6-diamidino-2-phenylindole dihydrochloride (DAPI; Sigma™). Slides were mounted and analyzed using a Zeiss Axio Imager M2 microscope™ equipped with the Apotome system (Zeiss™). The percentage of cells containing myofibrils and actin polygonal configuration was quantified by randomly counting at least 100 cells per experimental condition (Silva et al., 2006).

### Scanning electron microscopy (SEM)

HCs *T. cruzi*-infected or non-infected were treated for 72 h with the compounds at concentrations corresponding to the IC_50_ in single treatment and in combination (using the ratio 1:4 [Bz:AMD]). Then, they were fixed with 2.5% glutaraldehyde in 0.1 M Na-cacodylate buffer (pH 7.2) for 40 min at 25°C and postfixed with 1% OsO_4_, 0.8% potassium ferricyanide and 2.5 mM CaCl_2_ in the same buffer for 20 min at 25°C. The cells were dehydrated in an ascending ethanol series and dried by the critical point method with CO_2_, mounted on aluminum stubs, coated with an approximately 20 nm thick gold layer in a Sputter Coater 108 (Cressington Scientific Instruments) and examined on a Jeol JSM6390LV scanning electron microscope™ (Jeol, Tokyo, Japan) located in the Rudolf Barth Electron Microscopy Platform (Oswaldo Cruz Institute, Fiocruz, Rio de Janeiro, RJ, Brazil). Alternatively, the monolayer was gently scraped off with adhesive tape after the critical point method with CO_2_ as reported by de Lima et al., 2015.

### Transmission electron microscopy (TEM)

BT of *T. cruzi* and infected HCs were treated for 24 and 72 h, respectively, with the compounds at concentrations corresponding to the IC_50_ in monotreatment or in combination (using the ratio 1:4 [Bz:AMD]). Then, the cells were fixed and postfixed, as mentioned for the SEM analysis. The cells were dehydrated in ascending acetone and embedded in Polybed 812 resin™. Ultrathin sections were stained with uranyl acetate and lead citrate and examined with a JEOL 1200 EX transmission electron microscope™ (Jeol, Tokyo, Japan) located at the Centro Nacional de Biologia Estrutural e Bioimagem (CENABIO) at the Universidade Federal do Rio de Janeiro (UFRJ, Rio de Janeiro, Brazil).

### Statistical analysis

The obtained results are expressed as the mean ± SEM for each group from at least three independent experiments. The normality of the distribution of the variables was tested with Shapiro–Wilk test. Between-group comparisons were made using one-way ANOVA followed by Tukey’s *post-hoc* test, Kruskal-Wallis test followed by Dunn’s *post hoc* test or Student’s *t-*test (GraphPad InStat 8.0, GraphPad Software Inc. ™, La Jolla, USA). Values of *p*<0.05 were considered significant

### Ethics

All experimental protocols using animals to settle primary cardiac cell culture and to maintain and obtain *T. cruzi* blood forms were performed in accordance with Brazilian Law 11.794/2008 and regulations of the National Council of Animal Experimentation Control under license L038/2018 from the Ethics Committee for Animal Use of the Oswaldo Cruz Institute (CEUA/IOC).

## Results

### The additive interaction of Bz and AMD against the BT of *T. cruzi* and the ultrastructural analysis of the parasite phenotypic alterations caused by treatment

**Figure 2:**
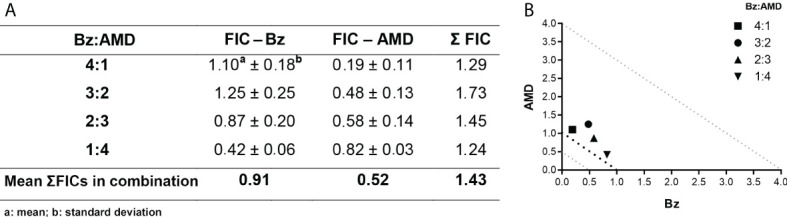
Effect of the combination Bz + AMD on BT forms of *T. cruzi* (Y strain) after 24 h of treatment, demonstrating the addictive interaction (0.5 < ΣFICs ≤ 4.0). **(A)** Table showing the mean ΣFICs of the interaction at the drug ratios tested; **(B)** Isobologram plotted with the FIC of AMD and Bz on the abscissa and the ordinate, respectively.

The trypanocidal activity of Bz_MT_ was significantly higher than that of AMD_MT_ against the BT of *T. cruzi*, as assessed by IC_50_/24 h values (8.82 ± 1.08 μM vs. 13.40 ± 1.26 μM, *p*=0.02). From these results, the concentrations of Bz and AMD were defined for the combined treatment assays (5:0, 4:1, 3:2, 2:3 and 1:4) using the fixed-ratio method (Fivelman et al., 2004). The pharmacological interaction of Bz and AMD on BT was classified as additive (no interaction) because the mean ƩFIC value was 1.43. All proportions tested were also classified as additive, with the best ratio of 1:4 (one part of Bz to four parts of AMD) with ƩFIC = 1.24 (Bz:AMD_Comb_ [Bz: IC_50_/24 h= 3.94; AMD: IC_50_/24 h= 15.00]) (**Figure 2**).

**Figure 3:**
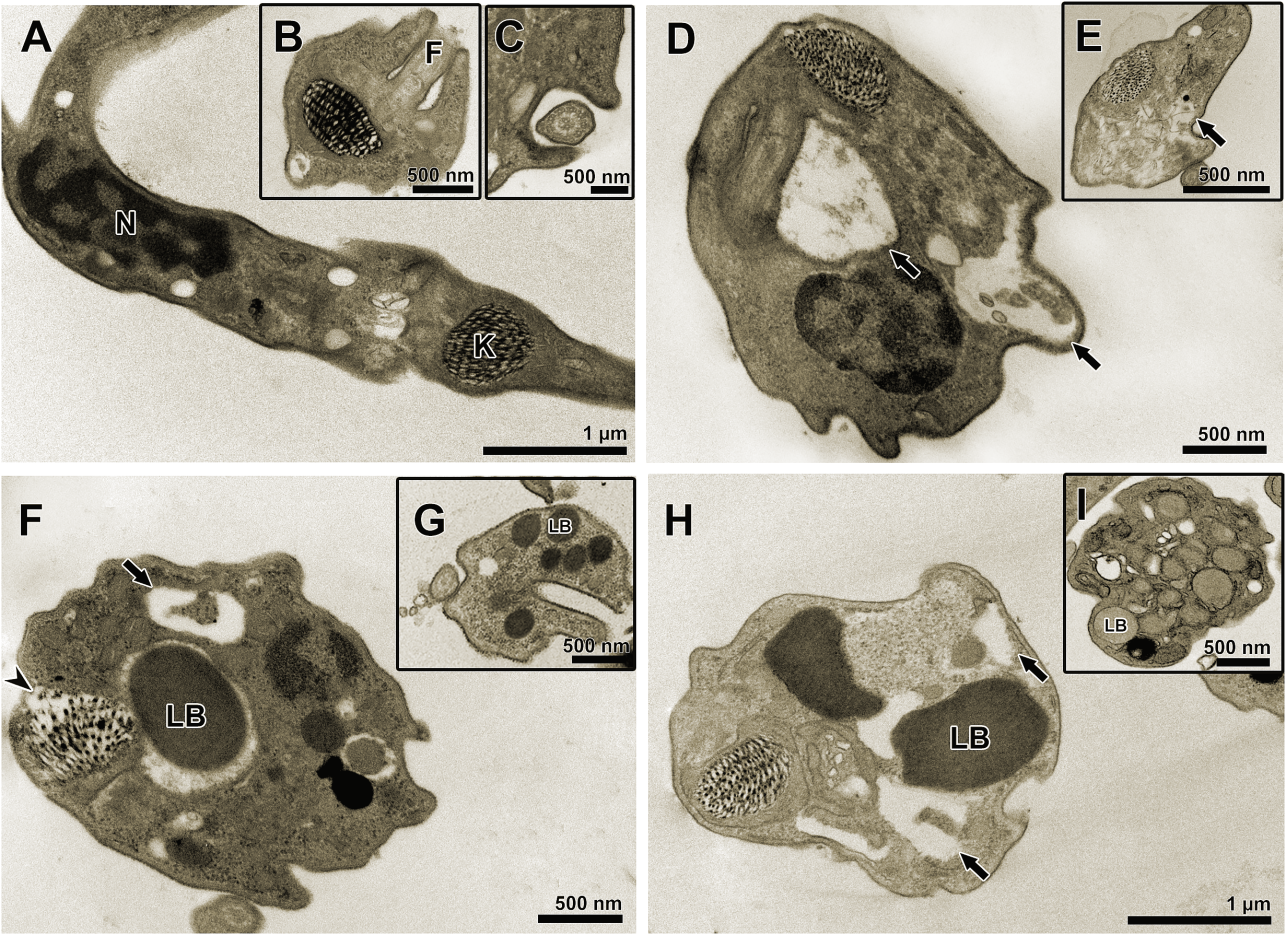
Transmission electron microscopy of *T. cruzi* bloodstream trypomastigotes treated for 24 h with Bz, AMD or their combination, showing the predominance of the phenotype generated by AMD in the combination Bz + AMD. **(A–C)** Untreated parasites exhibiting organelles with typical morphology; **(D, E)** Bz_MT_ (IC_50_/24 h = 8.82 μM); **(F, G)** AMD_MT_ (IC_50_/24 h = 13.40 μM) and **(H, I)** Bz:AMD_Comb_ (Bz: IC_50_/24 h = 3.94 μM; AMD: IC_50_/24 h = 15.0 μM). Black arrows indicate vacuolization and disorganization of the cytoplasm; arrowhead indicates disorganization of the kinetoplast. N, nucleus; K, kinetoplast; F, flagellum; LB, lipid bodies.

TEM was used to evaluate the ultrastructural phenotype of treated BT (**Figure 3**). Parasites treated with the three treatment regimens exhibited similar morphological changes, including vacuolization and disorganization of the cytoplasm (**Figures 3D–I**). However, in AMD_MT_ (**Figures 3F, G**) and Bz:AMD_Comb_(**Figures 3H, I**), the presence of lipid bodies was extensively observed.

### The cytotoxic effect of Bz and AMD on HCs and its additive trypanocidal effect on the intracellular forms of *T. cruzi*

**Figure 4:**
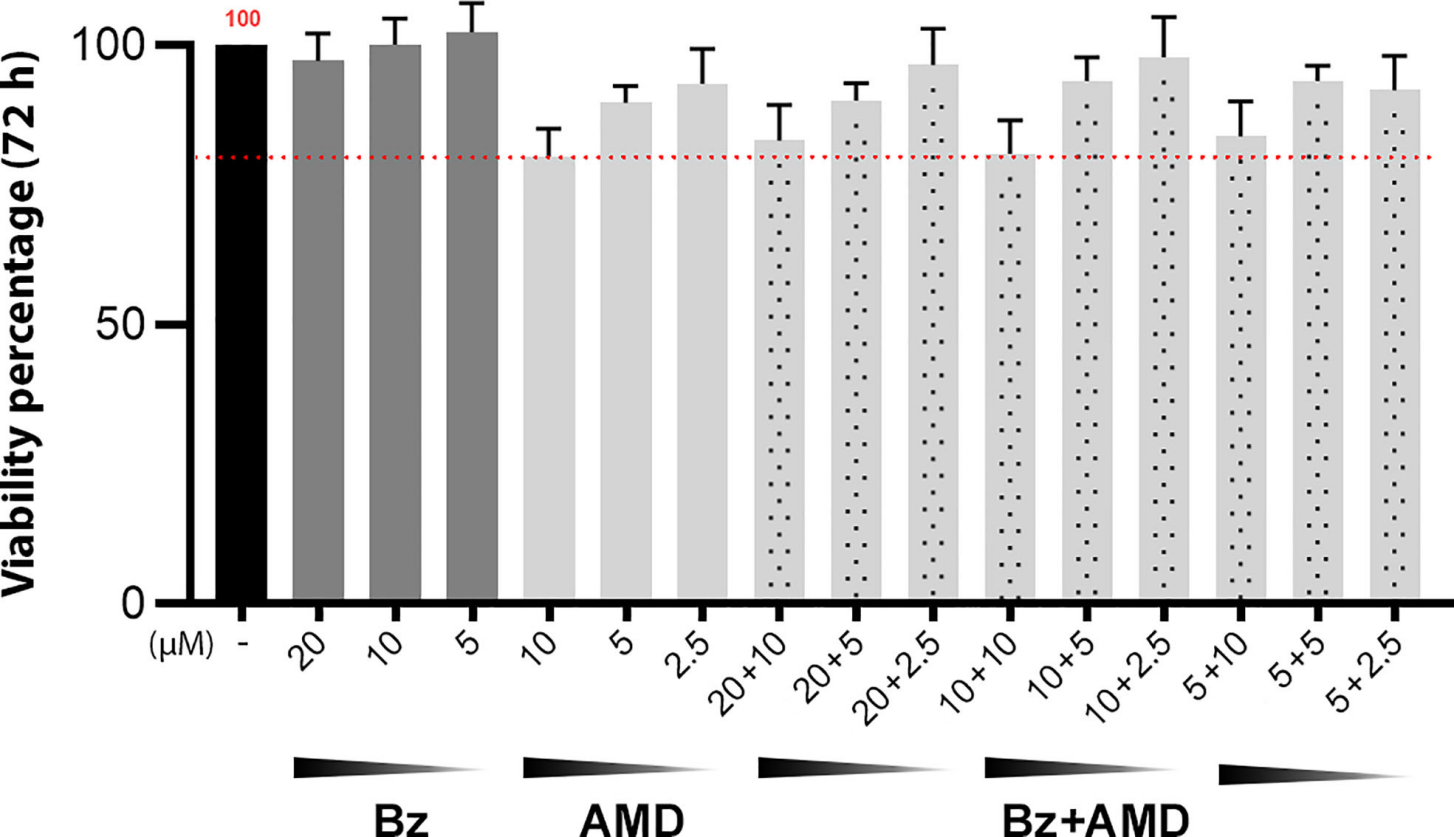
Viability of uninfected and treated HCs after 72 h of treatment with Bz, AMD or their combination, demonstrating the non-cytotoxicity of all treatment regimens. The black bar corresponds to the control condition (untreated); dark gray bars correspond to Bz and light gray bars to AMD, and the dotted bars correspond to the combined treatments. The red dotted line highlights 80% viability. (One-way ANOVA followed by Tukey’s *post-hoc* test).

To evaluate the trypanocidal effect of the combination Bz + AMD on the intracellular amastigote form of *T. cruzi* as well as a possible reversal of damage to the cytoskeleton of infected cells, primary cultures of HCs were used. To validate the concentrations of Bz and AMD tested, the viability of uninfected and treated HCs was evaluated using the PrestoBlue reagent. The viability of HCs in the Bz_MT_ group ranged from 97.2 to 102.3%, while for AMD_MT,_ the variation was 80.1-93.0%. However, all concentrations tested were considered nontoxic according to ISO 10993-5 (2009) (**Figure 4**).

**Figure 5:**
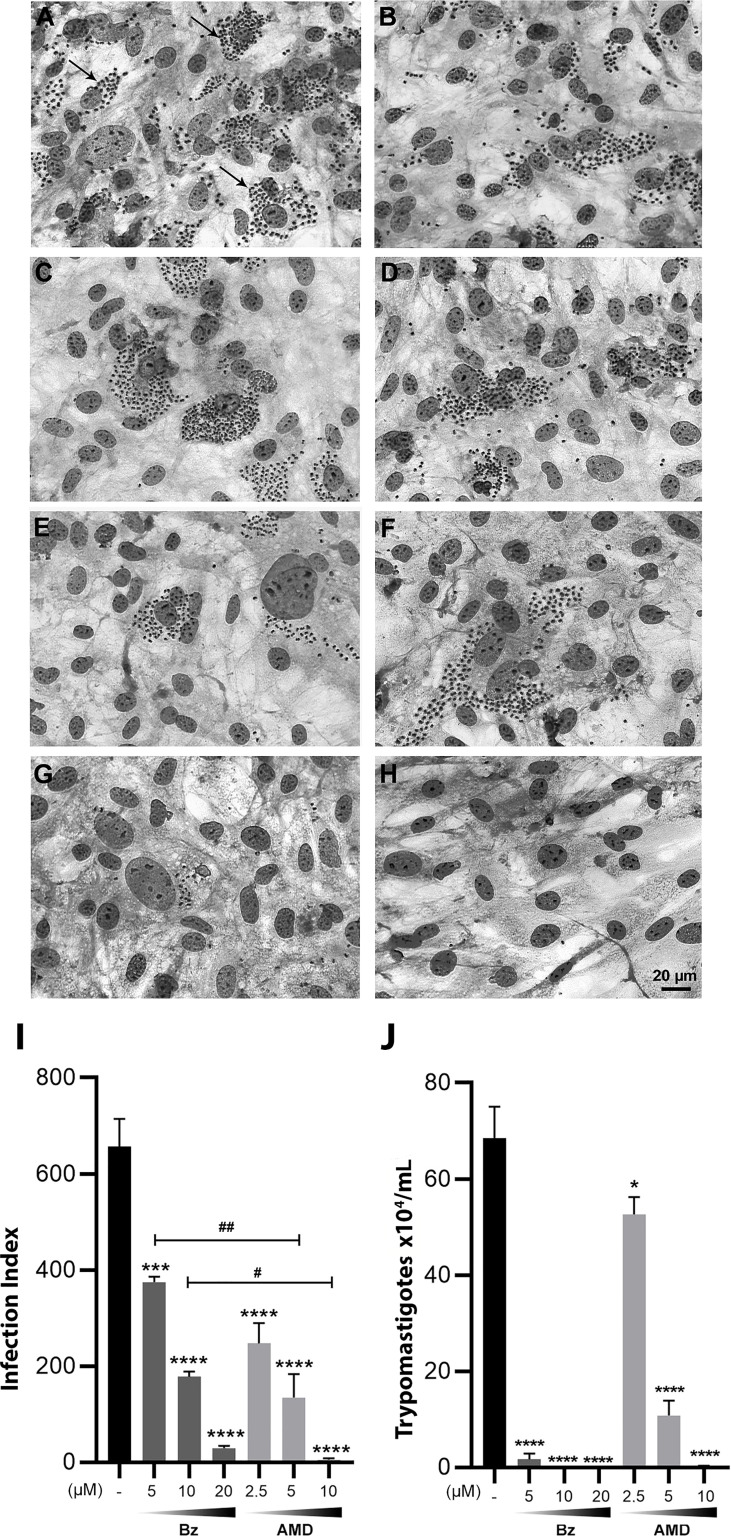
Trypanocidal effect of Bz and AMD in monotreatment on intracellular forms of *T. cruzi*-infected HCs, after 72 h of treatment, evidencing that AMD was more effective in eliminating intracellular parasites than Bz. Representative photomicrograph of infected HCs fixed and stained with Diff-Quick Staining: **(A, B)** Untreated cells (*Tc*); Cells treated with **(C)** 5 µM Bz; **(E)** 10 µM Bz; **(G)** 20 µM Bz; **(D)** 2.5 µM AMD; **(F)** 5 µM AMD; **(H)** 10 µM AMD; **(I)** infection index and **(J)** number of trypomastigotes in the supernatants. The black bar corresponds to the control condition (infected and untreated; dark and light gray bars correspond to Bz and AMD, respectively). Black arrows indicate intracellular parasites. All concentrations were compared to the control. *p* range: * ^#^: *p* < 0.05; ^##^: *p* < 0.01; ***: *p* < 0.001; ****: *p* < 0.0001. (One-way ANOVA followed by Tukey’s *post-hoc* test).

First, the IC_50_ of the infection index (II) for each drug in monotreatment was calculated after 24, 48 and 72 h of treatment (**Table 1**). After 24 and 48 h of treatment, no significant difference was observed in the trypanocidal activity between Bz_MT_ and AMD_MT_ (*p*>0.05). However, after 72 h of treatment, AMD_MT_ was more effective than Bz_MT_ in reducing the infection index (*p*=0.002) (**Figure 5**). The trypanocidal effect of each isolated compound was time-dependent, with the treatment of 72 h presenting the lowest IC_50_ values. Therefore, 72 h of treatment was set for the subsequent assays of combination.

**Table 1:** Trypanocidal effect of Bz and AMD in monotreatment on the intracellular forms of *T. cruzi*-infected HCs.

		IC_50_ II (µM)	
			
Treatment	24 h	48 h	72 h
**Bz_MT_**	>20	15.32^a^ ± 6.86^b^	5.37 ± 0.30
**AMD_MT_**	>10	7.36 ± 2.35	2.48 ± 0.23*

*: different from **Bz_MT_**; p range: *: p<0.05; **a**: mean; **b**: standard deviation

The obtained results are expressed as the IC_50_ of the infection index (II), calculated after 24, 48 and 72 h of treatment.

**Figure 6:**
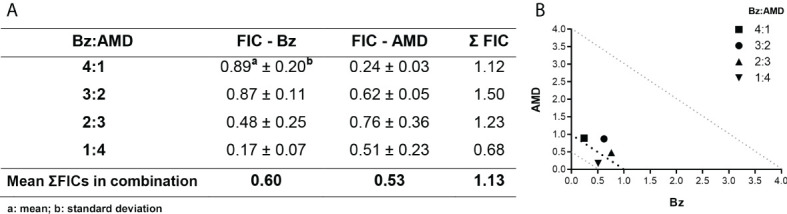
Effect of the combination Bz + AMD on intracellular forms of *T. cruzi*, after 72 h of treatment, demonstrating the addictive interaction (0.5 < ΣFICs ≤ 4.0). **(A)** Table showing the mean ΣFICs of the interaction at the drug ratios tested; **(B)** Isobologram plotted with the FIC of AMD and Bz on the abscissa and the ordinate, respectively.

The pharmacological interaction of Bz and AMD on intracellular forms of *T. cruzi* was classified as additive (ƩFIC = 1.13). All tested proportions were also classified as additive, with the best ratio of 1:4 (one part of Bz to four parts of AMD) with ƩFIC = 0.68 (Bz:AMD_Comb_ [Bz: IC_50_/24 h= 1.3 μM; AMD: IC_50_/24 h= 2.5 μM]) (**Figure 6**).

### The ultrastructural analysis of intracellular parasites in HCs and the phenotypical alterations caused by Bz and AMD treatment

**Figure 7:**
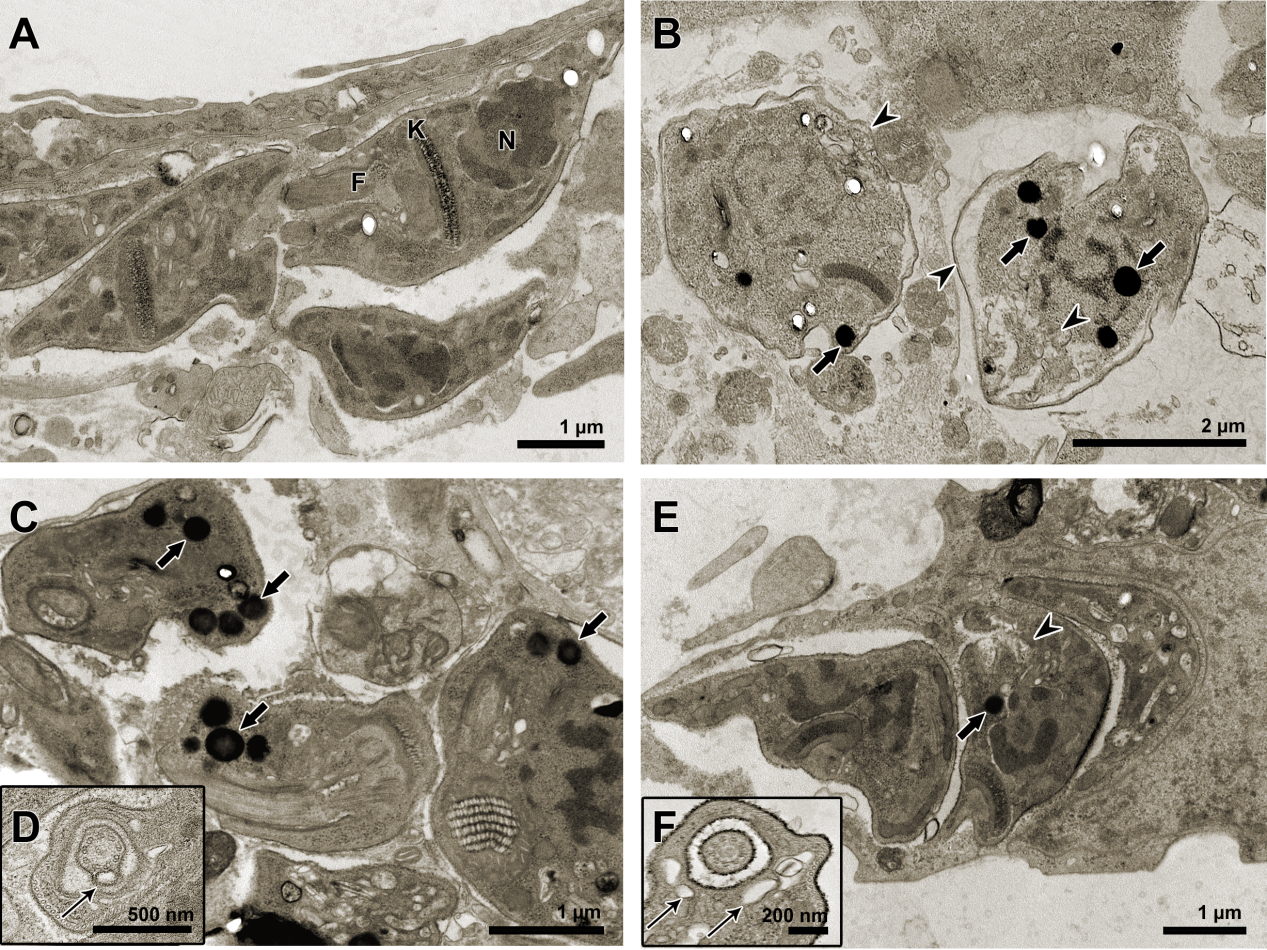
Transmission electron microscopy of *T. cruzi* intracellular forms treated for 72 h with Bz, AMD or their combination, showing the predominance of the phenotype generated by AMD in the combination Bz + AMD. **(A)** Untreated parasites exhibiting organelles with typical morphology; **(B)** Bz (IC_50_/72 h = 5.4 μM); **(C, D)** AMD (IC_50_/72 h = 2.5 μM) and **(E, F)** Bz:AMD (Bz: IC_50_/72 h = 1.3 μM; AMD: IC_50_/72 h = 2.5 μM). Black thin arrows indicate the presence of vesicles in the flagellum and flagellar pocket; black arrows indicate accumulation of lipid bodies; arrow heads indicate vacuolization and disorganization of the cytoplasm. N, nucleus; K, kinetoplast; F, flagellum.

TEM was also employed to evaluate the ultrastructural phenotype of intracellular parasites in HCs (**Figure 7**). Parasites treated with the three treatment regimens exhibited similar morphological changes, including the accumulation of lipid bodies and the formation of vacuoles in the cytoplasm (**Figures 7B–F**). Nonetheless, only in AMD_MT_ (**Figure 7D**) and Bz:AMD_Comb_ (**Figure 7F**) was the presence of vesicles in the flagellum and flagellar pocket detected.

**Figure 8:**
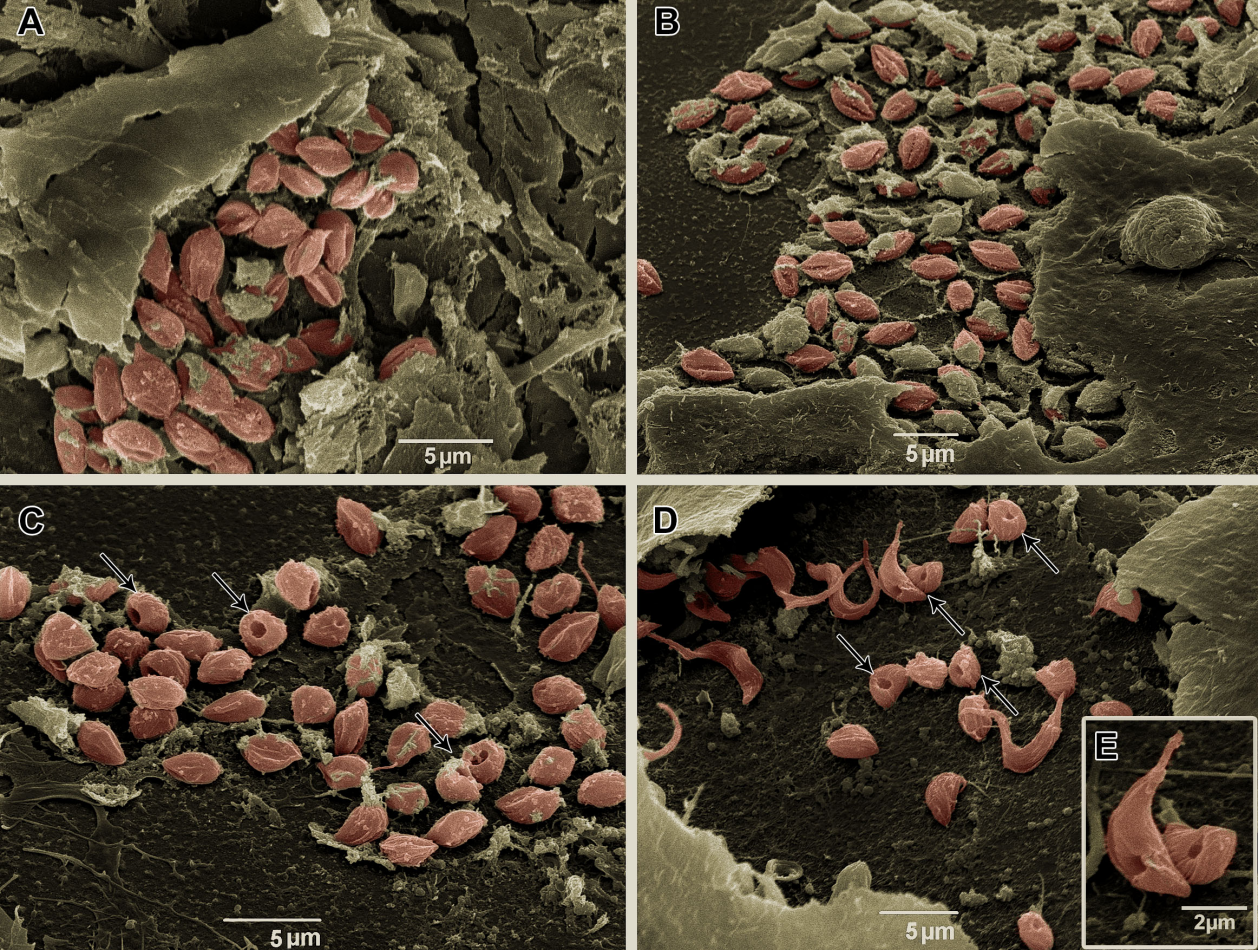
Scanning electron microscopy of *T. cruzi* intracellular forms treated for 72 h with Bz, AMD or their combination, showing the predominance of the phenotype generated by AMD in the combination Bz + AMD. **(A)** Untreated; **(B)** Bz_MT_ (IC_50_/72 h = 5.4 μM); AMD_MT_ (IC_50_/72 h = 2.5 μM) and **(D, E)** Bz:AMD_Comb_ (Bz: IC_50_/72 h = 1.3 μM; AMD: IC_50_/72 h = 2.5 μM). Parasites were digitally colored in red. Black thin arrows indicate dilatation of the flagellar pocket.

To visualize intracellular parasites by SEM, some of the samples of *T. cruzi*-infected HCs had their plasma membrane mechanically removed. No morphological differences were observed between untreated parasites (**Figure 8A**) and the Bz_MT_ group (**Figure 8B**). However, in AMD_MT_- (**Figure 8C**) and Bz:AMD_Comb_-treated *T. cruzi*-infected HCs, dilatation of the flagellar pocket was detected (**Figures 8D, E**).

### The reversal of damage to the cytoskeleton caused by *T. cruzi* infection generated by Bz and AMD treatment

**Figure 9:**
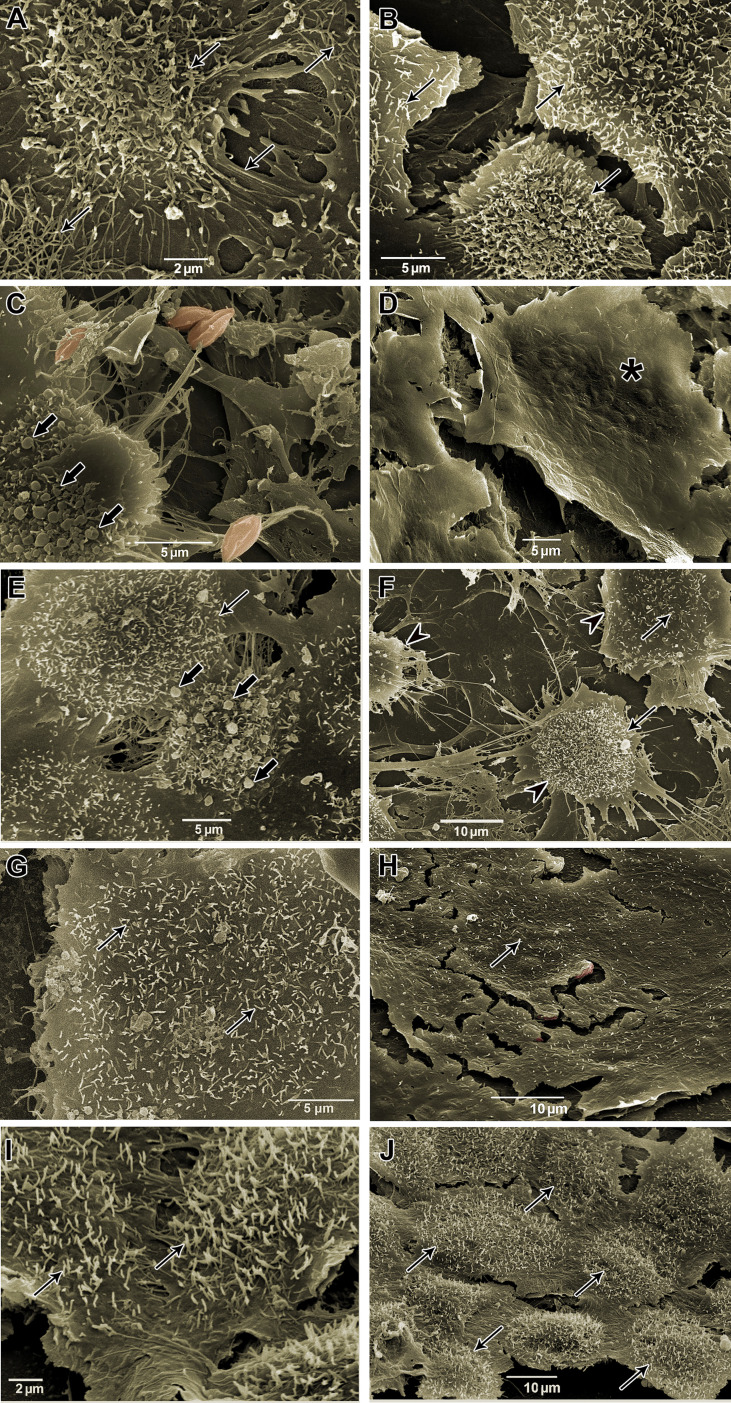
Scanning electron microscopy of *T. cruzi*-infected HCs treated for 72 h with Bz, AMD or their combination, demonstrating the predominance of the phenotype generated by Bz in the combination Bz + AMD. **(A, B)** Uninfected cells (NI); **(C, D)** Infected and untreated cells (*Tc*); **(E, F)** AMD_MT_ (IC_50_/72 h = 2.5 μM); **(G, H)** Bz_MT_ (IC_50_/72 h = 5.4 μM); **(I, J)** Bz:AMD_Comb_ (Bz: IC_50_/72 h = 1.3 μM; AMD: IC_50_/72 h = 2.5 μM). Parasites were digitally colored in red. Black thin arrows indicate cytoplasmic projections, similar to filopodia; arrow heads indicate cytoplasmic retraction; black arrows indicate apoptotic body-like vesicles, and asterisk shows surface protrusions compatible in size with the presence of intracellular forms.

Morphological analysis of *T. cruzi*-infected and treated HCs was also performed by SEM. Uninfected cells showed numerous projections, similar to filopodia, stretching out over the cell surface (**Figures 9A, B**). On the other hand, HCs infected with *T. cruzi* (144 hpi) showed numerous apoptotic body-like vesicles and areas with the absence of cytoplasmic projections (**Figures 9C, D**). After 72 h, in both groups, AMD_MT_ (**Figures 9E, F**) and Bz_MT_ (**Figures 9G, H**), the integrity of filopodia was partially reversed, especially in areas with a reduced number of intracellular forms. However, AMD_MT_ was less effective than Bz_MT_ in reversing the deterioration of the cytoarchitecture of infected HCs. Treatment with AMD maintained the presence of apoptotic body-like vesicles and pronounced cytoplasmic retraction (**Figures 9E, F**). In Bz:AMD_Comb_(**Figures 9I, J**) the predominance of the phenotype generated by Bz_MT_ was evidenced, with no structural changes.

**Figure 10:**
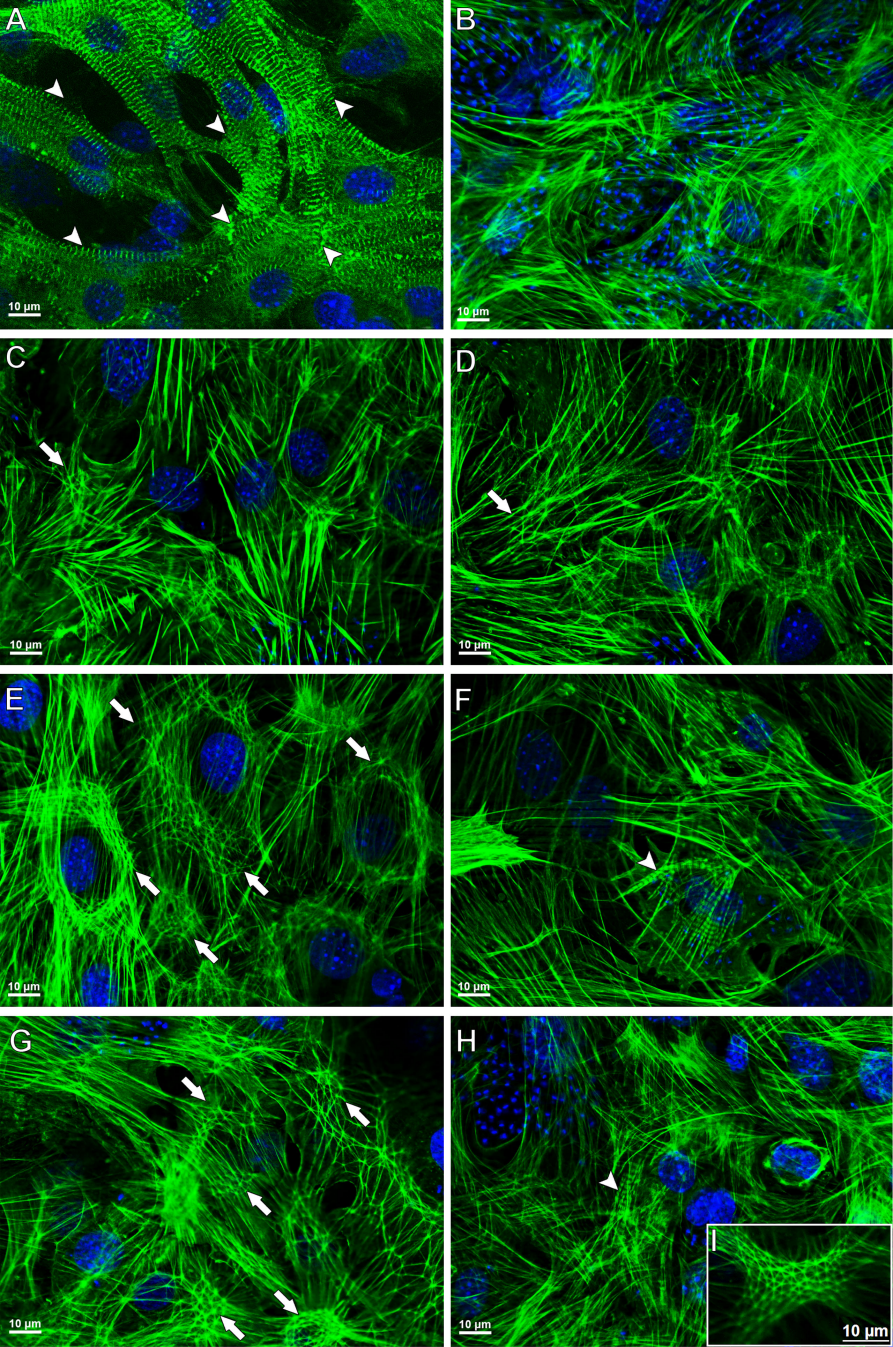
Cytoskeletal remodeling in *T. cruzi*-infected HCs treated for 72 h with Bz, AMD or their combination, demonstrating the predominance of the phenotype generated by Bz in the combination Bz + AMD. Actin filaments and DNA were detected by phalloidin–FITC and DAPI, respectively. **(A)** Myofibrils in uninfected cells (NI); **(B)** Myofibril disruption in untreated infected cells (*Tc*); **(C, D)** AMD_MT_ (IC_50_/72 h = 2.5 μM); **(E, F)** Bz_MT_ (IC_50_/72 h = 5.4 μM); **(G–I)** Bz:AMD_Comb_ (Bz: IC_50_/72 h = 1.3 μM; AMD: IC_50_/72 h = 2.5 μM). Of note, the rearrangement of actin structure in HCs is related to the reduction in intracellular parasite infection. Arrow heads indicate myofibrils; arrows indicate an actin polygonal configuration, and a thin arrow indicates an actin belt around the cell nucleus.

To investigate the effect of the combination Bz + AMD on the recovery of the cytoskeleton of *T. cruzi*-infected HCs, F-actin immunostaining was performed 72 h after the treatment (**Figure 10**). Disruption of myofibrils was observed after 144 hpi, while remodeling of the cytoskeleton was observed in all infected and treated groups (Bz_MT_, AMD_MT_ and Bz:AMD_Comb_), as shown by the myofibril architecture with an actin polygonal configuration interconnected by actin filaments such as an actin belt and an actin belt around the cell’s nucleus (**Figure 10**).

Furthermore, the percentage of cells containing myofibrils and actin polygonal configuration was calculated and is shown in **Table 2**. Our data demonstrated that in both Bz_MT_ and Bz:AMD_Comb,_ there was an increase in the percentage of cells containing myofibrils; however, this increase was not significant compared to infected and untreated HCs (cells containing myofibrils [%] =*Tc*: 0.89; Bz_MT_: 4.16 and Bz:AMD_Comb_: 2.67; *p*>0.05). Accordingly, all three treatments increased the percentage of cells containing the actin polygonal configuration. However, this was only significant in Bz_MT_ and Bz:AMD_Comb_ (cells containing myofibrils [%] =*Tc*: 0.89; Bz_MT_: 29.33 and Bz:AMD_Comb_: 27.25; *p <*0.05).

**Table 2:** Quantification of cytoskeletal remodeling in *T. cruzi*-infected HCs treated for 72 h with Bz and AMD in monotreatment or in combination.

(%) Cell containing	NI	*Tc*	Bz_MT_	AMD_MT_	Bz:AMD_Comb_
**Myofibrils**	70.60 ± 14.81^a^	0.89 ± 2.52^****^	4.16 ± 12.50^****^	1.41 ± 4.23^****^	2.67 ± 5.71^****^
**Actin polygons**	0.00 ± 0.00	0.89 ± 2.52	29.33 ± 25.52^** ##^	14.11 ± 17.08	27.25 ± 26.93^** ##^

HCs: uninfected (**NI**); infected and untreated (**Tc**); infected and treated with Bz (IC_50_/72 h = 5.4 μM); infected and treated with AMD (IC_50_/72 h = 2.5 μM) and infected and treated with Bz plus AMD (Bz: IC_50_/72 h = 1.3 μM; AMD: IC_50_/72 h = 2.5 μM). **a**: mean ± standard deviation. #: different from **Tc**; *: different from **NI**; p range: ** ##: p < 0.01; ****: p < 0.0001.

## Discussion

AMD is recognized by the US Food and Drug Administration (FDA) as a type III antiarrhythmic drug, according to the Vaughan Williams classification (Benaim et al., 2021). The repurposing of AMD was first thought to be an antifungal drug alternative (Courchesne, 2002). Then, the efficacy of AMD and its derivatives against the pathogenic trypanosomatids *Trypanosoma brucei, T. cruzi* and *Leishmania* spp. was demonstrated (Benaim et al., 2006; Serrano-Martín et al., 2009). In this study, we analyze the effects of the combination Bz + AMD, drugs with different mechanisms of action and pharmacokinetic profiles (Benaim et al., 2006; Wilkinson et al., 2011).

The activity of AMD on amastigotes of *T. cruzi* is related to the homeostatic disruption of Ca^2+^ and blockage of oxidosqualene cyclase activity, with this drug classified as an ergosterol biosynthesis inhibitor (EBI) (Benaim et al., 2006). On the other hand, Bz is a prodrug that requires activation to exert cytotoxic action. In trypanosomatids, this process involves reduction of the nitro group catalyzed by nitroreductases, which generate metabolites that interact with a wide range of biomolecules, especially DNA and thiols (Polak and Richle, 1978; Díaz de Toranzo et al., 1988; Wilkinson et al., 2011). In trypanosomatids, the nitro reduction mediated by nitroreductases does not involve oxygen and does not generate a significant level of oxygen consumption and free radical production. In contrast, in mammalian systems, Bz induces the production of reactive oxygen species (Wilkinson et al., 2011). Therefore, we hypothesized that the combination Bz + AMD could improve the efficacy of the etiologic treatment, especially in the chronic phase of CD, since these drugs are already approved for use in chronic patients (PCDT Chagas, 2018).

The combination of Bz and EBIs has been extensively investigated to improve their effectiveness for CD treatment. The combination of Bz with itraconazole, posaconazole, ketoconazole or fosravuconazole leads to a decrease in parasitemia and an increase in survival in *T. cruzi*-infected mice (Assíria Fontes Martins et al., 2015; Echeverría et al., 2020; da Araújo et al., 2000; Diniz et al., 2018). Moreover, the carvedilol, a beta-blocker widely used to treat cardiovascular diseases, such as AMD, also showed trypanocidal activity in both *in vitro* and *in vivo*, impairing the survival of trypomastigotes and reducing the whole-body parasite burden peak in infected mice (Rivero et al., 2021). In addition, the *in vitro* combination of AMD with itraconazole and posaconazole also cooperatively reduced infection rates and the multiplication of intracellular parasites (Benaim et al., 2006; Sass et al., 2019). However, Lourenço et al. (2018) failed to show improvements in the trypanocidal effect of Bz combined with AMD against epimastigotes of *T. cruzi* in comparison with Bz single treatment.

To our knowledge, this is the first study investigating the pharmacological interaction between Bz and AMD on the infective forms of *T. cruzi* blood trypomastigotes and intracellular forms. Our results reveal that the combination Bz/AMD is classified as an additive, indicating that there is no loss of the trypanocidal action of each compound when they are combined. Besides, even though the combination was not synergistic it was observed a reduction in the inhibitory concentration values of substances when combined. In addition, it was also noted that in both infective forms of *T. cruzi*, the ratio 1:4 for Bz:AMD_Comb_ was the closest to the synergistic effect (∑FIC ≤ 0.5) (Odds, 2003).

Furthermore, the AMD_MT_ was more effective in eliminating intracellular parasites than Bz_MT_. This result could be explained by the high susceptibility of the amastigotes to EBIs, which does not seem to be related to a higher sensitivity of the target enzymes but to a smaller pool of sterols in this intracellular stage of *T. cruzi* (Liendo et al., 1999; Urbina, 2001). In addition, the high lipophilicity of AMD can also favor the elimination of intracellular forms (Debbas et al., 1983; Madigan et al., 2019). Bz is more hydrophilic, and this chemical property is considered one of the reasons for its decreased curative effectiveness for chronic CD (Urbina, 2010; Molina et al., 2014).

We also investigated the impact of the combined treatment on the cytoarchitectural recovery of infected HCs. It has been widely reported that in HCs, *T. cruzi* (Y strain) infection induces a decrease in the expression and structural disorganization of cytoskeleton proteins, such as F-actin, α-actinin, vinculin, talin and paxillin, after 72 hpi (Melo et al., 2004; Melo et al., 2006; Melo et al., 2019). Furthermore, the microfilament destruction caused by the infection is one of the major factors contributing to cardiac arrhythmias due to the loss of transmission of the contractility force between cardiomyocytes (Pereira et al., 1993; Silva et al., 2006; Adesse et al., 2011). Analyzing the F-actin labeling of infected HCs, we found that Bz:AMD_Comb_led to a phenotype similar to the Bz_MT_, such as (i) formation of actin polygons, (ii) partial maintenance of the integrity of the striatum characteristic of the sarcomeric organization and (iii) perinuclear marking of F-actin. The formation of polygonal structures formed by short actin filaments connected to nucleation centers has been extensively documented as characteristic of the reorganization process of this protein, being a crucial step in myofibril structure recovery and the contractile capacity of cardiomyocytes (Lin et al., 1989; Silva et al., 2006; Adesse et al., 2011). The polymerization of F-actin begins with the nucleation of protein monomers, which is mainly regulated by the actin-related protein complex (ARP). This ARP also allows lateral binding between actin filaments, generating a branched network with a polygonal aspect. The formation of this microfilament network allows the association of the cytoskeleton with the plasma membrane, forming filopodia and lamellipodia on the cell surface (Alberts et al., 2002). Thus, our results suggest that the structural disorganization of F-actin may be related to the absence of filopodia in infected and untreated HCs (144 hpi). Therefore, as observed with the F-actin labeling assays, the SEM analysis revealed that Bz:AMD_Comb_ led to a predominance of the phenotype generated by Bz_MT_ with partial maintenance of the integrity of long and numerous filopodia and of cytoplasmatic projections. We suggest through both analyses that the morphological recovery of the infected HCs treated with Bz:AMD_Comb_is associated with the elimination of intracellular parasitism.

Assessing the morphology of BT and intracellular parasites by electron microscopy, we also observed the predominance of the phenotype generated in AMD_MT_ in the Bz:AMD_Comb_ group, such as the formation of cytoplasmic lipid bodies and dilation in the flagellar pocket. These morphological changes have already been described in amastigotes and epimastigotes treated with AMD (Adesse et al., 2011; Veiga-Santos et al., 2012). Taken together, these results suggest that in the Bz:AMD_Comb_mode of action of AMD, it was not disturbed by the presence of Bz.

In conclusion, this study demonstrated that the combination of Bz and AMD did not interfere with the trypanocidal efficacy of each drug alone against the relevant parasite forms for mammalian host infection. Moreover, although the combination with Bz did not increase the trypanocidal effect of AMD, the combined treatment of *T. cruzi*-infected HCs seems to exert a cardioprotective effect because it was more effective in recovering the damage to the host cell cytoskeleton. Further studies are under way to investigate the effect of the Bz/AMD combination in experimental CCC.

## Data availability statement

The original contributions presented in the study are included in the article/supplementary material. Further inquiries can be directed to the corresponding author.

## Ethics statement

The animal study was reviewed and approved by Comissão de Ética no Uso de Animais do Instituto Oswaldo Cruz (CEUA-IOC).

## Author contributions

JB contributed to conception and design of the study, organized the database, performed the statistical analysis, wrote the first draft of the manuscript. YP-R, LP, AD, and KS contributed to conception and design of the study. TM contributed to conception and design of the study, wrote sections of the manuscript. HB, JL-V, and SC wrote sections of the manuscript.

All authors contributed to manuscript revision, read, and approved the submitted version

## Funding

The authors are also grateful to Fundação de Amparo à Pesquisa do Rio de Janeiro (FAPERJ, BR), Coordenação de Aperfeiçoamento de Pessoal de Nivel Superior (CAPES, BR), Conselho Nacional de Desenvolvimento Científico e Tecnológico (CNPq, BR) and Fundação Oswaldo Cruz (FIOCRUZ, BR) for financial support and fellowships.

## Acknowledgments

The authors thank the Plataforma de Microscopia Eletrônica Rudolf Barth at Instituto Oswaldo Cruz, Fiocruz; and Daniel Gonçalves Lucif Vieira from Centro Nacional de Biologia Estrutural e Bioimagem (CENABIO) at the Universidade Federal do Rio de Janeiro.

## Conflict of interest

The authors declare that the research was conducted in the absence of any commercial or financial relationships that could be construed as a potential conflict of interest.

## Publisher’s note

All claims expressed in this article are solely those of the authors and do not necessarily represent those of their affiliated organizations, or those of the publisher, the editors and the reviewers. Any product that may be evaluated in this article, or claim that may be made by its manufacturer, is not guaranteed or endorsed by the publisher.
